# Research into the functional components and antioxidant activities of North China rice wine (Ji Mo Lao Jiu)

**DOI:** 10.1002/fsn3.39

**Published:** 2013-06-07

**Authors:** Shuai He, Xiangzhao Mao, Pei Liu, Hong Lin, Zuyuan Du, Ning Lv, Jichen Han, Cuifang Qiu

**Affiliations:** 1 College of Food Science and Engineering, Ocean University of ChinaQingdao, 266003, China; 2 Shandong Ji Mo Lao Jiu winemaking companyQingdao, 266200, China; 3 Shandong Entry-Exit Inspection and Quarantine BureauQingdao, 266001, China

**Keywords:** Amino acids, antioxidant activities, Chinese rice wine, mineral elements, oligosaccharides, total phenols

## Abstract

Over the last decade, considerable experimental evidence has supported the view that grape wine and South China rice wine are rich in diverse nutrients and have powerful antioxidant activity. However, little research has been carried out for North China rice wine, of which Ji Mo Lao Jiu (JMLJ) is the outstanding representative. In this study, the functional components and antioxidant activity of JMLJ were investigated. Twenty-eight free amino acids were found in JMLJ, much more than that previously reported in other Chinese rice wines (16–21). Functional oligosaccharides (5290.222 mg/L), total phenols (722.431 ± 10.970 mg/L), and mineral elements (9) were rich in JMLJ. When compared with synthetic antioxidants, such as butylated hydroxyanisole (BHA) and butylated hydroxytoluene (BHT), JMLJ showed effective 1,1-diphenyl-2-picryl-hydrazyl (DPPH) radical scavenging and reducing capacity. The results of this study lay the foundation for promoting the utilization of JMLJ and the development of North China rice wine in the food industry.

## Introduction

As a traditional alcoholic beverage, Chinese rice wine has been popular in China for centuries. It is produced by the fermentation of traditional rice wine starters (wheat Qu or rice Qu) by beneficial microbes, and has been widely known for its healthcare functions (Que et al. 2006a), which is related to the antioxidant activity and rich content of various functional components.

Regarding the difference in geographical location and cereal materials, for instance, sticky rice in South China, millet and wheat in North China, Chinese rice wine can be divided into South China rice wine and North China rice wine. Recently, increasing evidence has highlighted that South China rice wine is rich in a variety of functional components, such as free amino acids (Shen et al. 2010), oligosaccharides (Yu et al. 2003, Guang-fa 2008), phenol compounds (Que et al. 2006a), etc., together with strong antioxidant activities (Que et al. 2006a). However, similar research has rarely been reported for North China rice wine.

As reported previously (Xie et al. 2004), Chinese rice wine is similar to or even better than red wine in terms of health functions. Therefore, we expected that Ji Mo Lao Jiu (JMLJ), one of the most important rice wines of North China, should possess some functional components and antioxidant activity, which at least partly contribute to its healthy effects. The main objective of this study was to determine the contents of free amino acids, functional oligosaccharides, total phenols, and mineral components, and to evaluate the antioxidant properties and antioxidant activities with methods of scavenging activity of 1,1-diphenyl-2-picryl-hydrazyl (DPPH) radicals and reducing capacity, respectively. This work serves as an important basis for further study.

## Materials and Methods

### Reagents

DPPH, 3-(2-pyridyl)-5,6-bis(4-phenyl-sulfonic acid)-1,2,4-triazine (Ferrozine), a-tocopherol, butylated hydroxyanisole (BHA), butylated hydroxytoluene (BHT) were purchased from Sigma Chemical Co. (St. Louis, MO). All other chemicals used were of analytical grade.

### Wine samples

Original rice wine of 2011 vintage (JMLJ) was provided by Shandong Ji Mo Lao Jiu winemaking company.

### Determination of functional components

#### Free amino acids

The amino acid composition of rice wine was determined by high-performance cation-exchange chromatography with postcolumn ninhydrin derivatization and Spackman's achievement (Spackman et al. 1958). After pretreatment with ethanol precipitation to remove proteins, the sample was blow-dried by a nitrogen blowing instrument and then was redissolved using the sample diluents (pH 2.2, 0.12 N, citrate solution).

A Sykam S-433D automatic amino acid analyzer was equipped with Sykam S 2100 Quaternary gradient pump, S 4300 Amino Acid Reaction Module, and S 5200 autosampler (SYKAM GmbH, Munich, Germany). Separation was performed on a high-efficiency sodium cation separation column LCA K07/Li Peek column (4.6 × 150 mm). The experimental conditions were as follows: the mobile phase consisted of buffer A (lithium citrate buffer A-1, pH 2.90), buffer B (lithium citrate buffer B-1, pH 4.20), buffer C (lithium citrate/borate buffer C-4, pH 8.00), and buffer D (regenerative liquid, volume of isopropanol/ultrapure water = 30/70); the gradient elution was implemented as follows: A:B:C:D = 100:0:0:0 from start to 10 min; A:B:C:D = 79:21:0:0 from 11 to 30 min; A:B:C:D = 62:38:0:0 at 41 min; A:B:C:D = 0:100:0:0 from 63 to 66 min; A:B:C:D = 0:0:100:0 from 74 to 84 min; A:B:C:D = 0:0:86:14 from 87 to 90 min; A:B:C:D = 0:0:78:22 at 90.01 min; A:B:C:D = 0:0:76:24 at 107 min; A:B:C:D = 0:0:0:100 from 107.01 to 112 min; A:B:C:D = 100:0:0:0 from 112.01 to 135.9 min; the flow rate of the mobile phase was 0.45 mL/min and the flow rate of the derivatizing reagent was 0.25 mL/min; the column temperature gradient program was 38°C from start to 38 min, 60°C from 48 to 80 min, 74°C from 101 to 114 min, and 38°C from 122 to 135.5 min; the postcolumn reaction equipment was kept at 130°C; the detector was an integrated dual-wavelength spectrophotometer; the temperature of the autosampler was kept at 5°C and the injection volume was 25 μL for both standard and samples.

#### Oligosaccharides

Oligosaccharides were examined by the method of HPAEC-IPAD (high-performance anion-exchange chromatography with integrated pulsed amperometric detection) (Yu et al. 2003). The rice wine was pretreated by the ion chromatography pretreatment column Cleanert IC-RP (Agela Technologies, Tianjin, China) to remove the proteins. The treated rice wine was then analyzed with ICS3000 from Dionex (Qingdao Institute of Bioenergy and Bioprocess Technology, Qingdao, China), consisting of a GS50 gradient pump with an AS autosampler, an AS thermal compartment, and an ED50 electrochemical detector equipped with a thin-layer-type amperometric cell comprising a gold electrode, a combination glass and Ag/AgCl (3 mol/L KC1) reference electrode, and a titanium counter electrode consisting of the cell body. Separation was performed on an AminoPac PA 10 column set comprising a guard column (50 × 2 mm) and an analytical column (250 × 2 mm). The columns and the electrochemical detection cell were placed inside the AS50 thermal compartment for temperature control. Chromatographic system control, data acquisition, and analysis were performed by means of Chromeleon Software (Dionex).

#### Total phenols

Total phenols were determined with the Folin-Ciocalteu reagent according to the method of Slinkard and Singleton (1977), using hydrated gallic acid as standard with some modifications. 150 μL of rice wine and 1.25 mL of Folin-Ciocalteu reagent were added into a 25 mL colorimetric tube. After full mixing, 3.75 mL of 200 g/L Na_2_CO_3_ was added and the reaction system was made up to 25.0 mL. The mixture was then allowed to stand at 30°C for 2 h after intermittent shaking. Finally, the absorbance at 760 nm was measured with a spectrophotometer. The concentration of total phenolic compounds was determined using an equation obtained from a standard hydrated gallic acid graph:


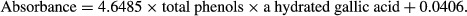


#### Mineral elements analysis by ICP-MS

The method used in the detection of mineral components is based on Valerije Vrček and Vinković Vrček (2012) and Herwig et al. (2011) with modifications. An Agilent Technologies 7500ce ICP-MS system (Agilent, Inc., Qingdao, China), which utilizes an octopole collision/reaction cell, was operated in this study.

The sample was digested as follows. First, about 0.5 g of sample was weighed in a polytetrafluoroethylene digestion tank, and 1.0 mL of H_2_O_2_ and 3.0 mL of concentrated HNO_3_ were added to disperse the sample homogeneously. Second, the sample was left overnight and the digestion tank was supplemented with 1.0 mL of concentrated HNO_3_. Third, the reaction solution was deposited in a microwave system (MARS EXPRESS, CEM, Qingdao, China). The program of sample digestion is as follows: the temperature elevation time from room temperature to 120°C was 6 min with a holding time of 4 min; from 120°C to 160°C it was 6 min with a holding time of 6 min; from 160°C to 185°C it was 8 min with a holding time of 20 min. After cooling, the digests were quantitatively transferred into a 50.0 mL volumetric flask and made up to volume with ultrapure water obtained from a Milli-Q water purification system (Millipore, Belford, MA).

### Determination of antioxidant activities

#### DPPH radical scavenging activity

Free radical scavenging activity was measured with the methods based on the reduction of DPPH (Shimada et al. 1992) with slight modifications. Three milliliters of 0.1 mmol/L DPPH radicals dissolved in ethanol was added to 0.2 mL of water solution containing different volumes of rice wines (50, 100, and 200 μL). The evenly mixed solution was placed in the dark. After 30 min, absorbance was measured at 517 nm. Lower absorbance of the reaction mixture indicated higher free radical scavenging activity. The scavenging of DPPH radicals was calculated by the following equation:


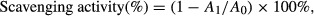


where *A*_0_ is the absorbance of the control reaction and *A*_1_ is the absorbance in the presence of rice wine. 50, 100, 200 μL of 1 g/L BHA and 1 g/L BHT were used as positive controls.

#### Reducing capacity

The reducing capacity of rice wine was determined by the method of Oyaizu (1986) with slight modifications. Different volumes (20, 50, 100, and 200 μL) of rice wines dissolved in 1 mL of distilled water were mixed with phosphate buffer (2.5 mL, 0.2 mol/L, pH 6.6) and potassium ferricyanide (2.5 mL, 10 g/L). The mixtures were incubated at 50°C for 20 min and aliquots (2.5 mL) of trichloroacetic acid (100 g/L) were added, after which the mixtures were centrifuged at 1000 *g* for 10 min. The upper layer of solution (2.5 mL) was mixed with distilled water (2.5 mL) and FeCl_3_ (0.5 mL, 1 g/L), and then the absorbance was measured at 700 nm in a UV–VIS spectrophotometer. In general, the greater absorbance value indicates the stronger reducing power. 20, 50, 100, 200 μL of 1 g/L BHA and 1 g/L BHT were determined as positive controls.

### Statistical analyses

Most of the experiments were conducted in triplicate. The average value and standard deviation (SD) were calculated and expressed.

## Results and Discussion

### Free amino acids

The free amino acid composition of JMLJ is presented in Table 1, which revealed that a total of 28 amino acids (1264.516 mg/L) were recorded in JMLJ by ion-exchange chromatography with postcolumn derivatization using ninhydrin as derivatization agent and ultraviolet (UV) detection.

**Table 1:** Free amino acid concentrations (mg/L) in JMLJ

Amino acids	Content	Amino acids	Content
Lysine^1^	47.884	Methionine^1^	21.736
Threonine^1^	21.338	Isoleucine^1^	19.867
Valine^1^	41.671	Leucine^1^	78.505
Phenylalanine^1^	41.073	Tryptophane^1^	2.590
Aspartic acid^2^	7.786	Serine^2^	38.990
Alanine^2^	166.536	Histidine^2^	10.117
Tyrosine^2^	35.933	Glycine^2^	43.847
Glutamic acid^2^	119.996	Arginine^2^	80.563
Cystine^2^	10.010	Asparagine^2^	89.380
Proline^2^	127.717	Citrulline^3^	5.861
Ornithine^3^	52.693	β-Alanine^3^	1.027
γ-Aminobutyric acid^3^	172.391	Phosphoethanolamine^3^	3.223
α-Aminobutyric acid^3^	0.297	Taurine^3^	1.487
β-Aminoisobutyric acid^3^	8.768	Phosphoserine^3^	13.230
Total α-amino acids (TAA)	1264.516		
Essential α-amino acids (EAA)	274.664(EAA/TAA = 0.217)		
Nonessential α-amino acids (NEAA)	730.875(NEAA/TAA = 0.578)		
Nonprotein α-amino acids/(NPAA)	258.977(NPAA/TAA = 0.205)		

JMJL, Ji Mo Lao Jiu.

1 Essential amino acids.

2 Nutritionally nonessential amino acids.

3 Nonprotein amino acids.

Essential amino acids (EAA) are those that cannot be synthesized in adequate amounts by the organism, which must be provided from the diet to meet optimal needs under conditions where utilization rates are greater than synthesis (Wu 2009). As shown in Table 1, all the eight EAA (in different proportions) were determined, the total of which accounted for 21.7% of total amino acids (TAA). Leucine and lysine were found in appreciable amounts in JMLJ.

Of the more than 300 AA in nature, only 20 (α-AA) serve as building blocks of proteins. However, nonprotein amino acids (NPAA; e.g., ornithine, citrulline, and homocysteine) and non-α-amino acids (NAA; e.g., taurine and β-alanine) also play important roles in cell metabolism (Manna et al. 2009). It can be observed that γ-aminobutyric acid (GABA, one of the most important NPAA) accounted for the maximum share (172.391 mg/L) of all the free amino acids detected in JMLJ. GABA was speculated to be produced by α-decarboxylation reaction of glutamate decarboxylase from glutamate (Kaufman et al. 2006), which was the highest concentration of all detected amino acids in millet (Lorenz et al. 1980), the raw material of JMLJ. Studies had shown that GABA possessed a variety of biological activities, such as reducing blood pressure (DeFeudis 1983), adjusting arrhythmia (Okada 2001), regulating hormone secretion (Linli and Bilige 2007), anxiolytic activity (Sasaki et al. 2002), improving sleeping quality (Kayahara and Sugiura 2001), and airway hyper-reactivity in asthmatic mice (Xu et al. 2009). These properties of GABA clearly indicate that JMLJ can be used in the field of functional foods.

It was shown that ornithine has various biological functions, such as protecting the liver and treating liver diseases by producing amino acid infusion (Wang et al. 2007); therefore, it is utilized in the food and medical industries. From Table 1, we can find that ornithine is another important NPAA in JMLJ, with a content of 52.693 mg/L, which contributed to the biological function of JMLJ in protecting human liver.

### Isomalto-oligosaccharides

Most studies involving functional oligosaccharides have been carried out using inulin-based fructose oligosaccharides (FOS), together with various forms of galacto-oligosaccharides (GOS) (Macfarlane et al. 2007). The functional composition status and physiological function of these compounds have been extensively studied (Gibson et al. 2004; Roberfroid 2007). A variety of other potentially functional compounds such as isomalto-oligosaccharides (IMOs) have also been identified. Yang and Tsai (1993) and Chen et al. (2001) found that IMOs could significantly improve the frequency of spontaneous defecation, decrease plasma total lipids, cholesterol and triglyceride concentrations, and increase calcium or phosphorus absorption as well.

The present study indicated that JMLJ was rich in IMOs (5290.222 mg/L), including isomaltose, isomalto trisaccharide, and panose. The principal IMO was isomaltose (3550.017 mg/L) (Table 2) followed by panose (1625.986 mg/L). It is well documented that isomaltose is a disaccharide with a low sweetness (Miyake et al. 1985), and isomaltose hypgather can significantly improve the immune function of soft-shelled turtles (Kai et al. 2010). Panose is another important IMO with bifidogenic activities in vitro. It can significantly enhance the growth of *Bifidobacterium*, and reduce the growth of *Bacteroides* and also *Clostridium* to some extent (Mäkeläinen et al. 2009). This study showed that JMLJ had important physiological functions and was especially good for the aged. Therefore, regular and moderate drinking of JMLJ will benefit people's health.

**Table 2:** The concentrations (mg/L) of IMOs in JMLJ

	Isomaltose	Isomaltotrisaccharide	Panose	Total content
JMLJ	3550.017	114.219	1625.986	5290.222

IMOs, isomalto-oligosaccharides; JMJL, Ji Mo Lao Jiu.

### Total phenols

It is widely known that phenolic compounds have a protective action on the organism against cardiovascular and degenerative diseases (Heim et al. 2002; Huisman et al. 2004), and have been viewed as powerful in vitro antioxidants (Que et al. 2006a), antimicrobial, and anti-inflammatory substances (Kuo et al. 2011). The antioxidant activity of phenolic compounds of wine and beer has been clearly reported. Estruch (2000) established the protective effect of wine and other alcoholic beverages on coronary heart disease and proved that wine polyphenols had strong antioxidant activity, while Ghiselli et al. (2000) showed that the polyphenols in beer could enhance human plasma antioxidant activity. A few similar studies have been carried out for rice wine, especially North China rice wine.

In this study, 722.431 ± 10.970 mg of phenolic compounds was detected in 1 L of JMLJ, which was much higher than that of white wine (420.0–452.4 mg/L) and beer (333.0–357.2 mg/L) (Gorinstein et al. 2000). It is hypothesized that the phenolic compounds detected in JMLJ are derived almost exclusively from millet when compared with red wine, of which polyphenols are produced from grapes (Que et al. 2006a).

### Mineral elements

Humans require more than 22 mineral elements, which can all be supplied by an appropriate diet (White and Broadley 2005). However, people who subsist on cereals or hold an imbalanced dietary structure, often lack some trace elements (TEs), such as iron (Fe), zinc (Zn), calcium (Ca), magnesium (Mg), copper (Cu), or selenium (Se). Many experimental and clinical studies on selenium evidenced that Se possesses a strong bioactivity and is of major importance to human health (Tanguy et al. 2012). Adequate dietary selenium intake is needed to prevent the occurrence of diseases such as prostate and colorectal cancer (Peters and Takata 2008). It was also indicated that other TEs, for example, copper (Cu) and zinc (Zn) are essential for plant growth and human and animal nutrition (Singh et al. 2011).

This study determined four constant elements and five essential TEs, namely potassium (K), sodium (Na), Ca, Mg and Fe, Zn, manganese (Mn), Cu, and Se (Table 3). In terms of constant elements, the amount of K was found to be the largest (667.430 mg/kg) followed by Mg (305.578 mg/kg), Na (199.004 mg/kg), and Ca (167.231 mg/kg). The concentration of Fe was the largest (9.925 mg/kg) among all the five TEs detected in JMLJ. All these results indicate that JMLJ exhibited strong biological activities and physiological function in terms of the rich mineral elements.

**Table 3:** The contents (mg/kg) of mineral elements in JMLJ

	Constant elements				Trace elements				
		
Mineral elements	K	Mg	Na	Ca	Fe	Zn	Mn	Cu	Se
	667.43	305.578	199.004	167.231	9.925	3.94	3.275	0.119	0.009

JMJL, Ji Mo Lao Jiu.

### Antioxidant activity

Antioxidant activities of red wine (Surh et al. 1999; De Beer et al. 2003), beer (Wei et al. 2001), and South China rice wine (Que et al. 2006b) have been clearly reported. This study evaluated the antioxidant activities of JMLJ with methods of scavenging activity of DPPH radicals and reducing capacity.

#### DPPH radical scavenging activity

Scavenging DPPH radicals is a widely used method to determine antioxidant activity in a relatively short time compared with other methods (Que et al. 2006a). The reduction capability of DPPH radicals was determined by the decrease in the absorbance at 517 nm, which is induced by antioxidants (Gülçın et al. 2003). This study used 1 g/L BHA and 1 g/L BHT as positive controls.

**Figure 1:**
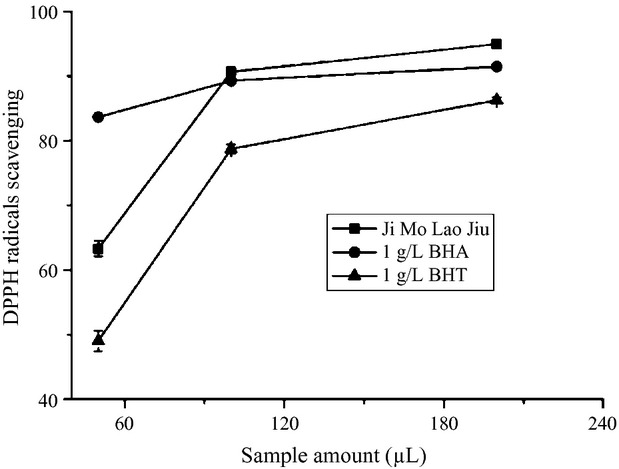
1,1-Diphenyl-2-picryl-hydrazyl (DPPH) radical scavenging activity of Ji Mo Lao Jiu, butylated hydroxyanisole (BHA), and butylated hydroxytoluene (BHT).

JMLJ showed DPPH radical scavenging activity at all amounts (Fig. 1). The DPPH radical scavenging effects of rice wine and positive controls decreased in the order of JMLJ>BHA>BHT, and were 94.95 ± 0.14%, 91.47 ± 0.22%, and 86.28 ± 0.41% at the amount of 200 μL, respectively. These results prove that JMLJ exhibited a high radical scavenging activity in vitro.

#### Reducing capacity

**Figure 2:**
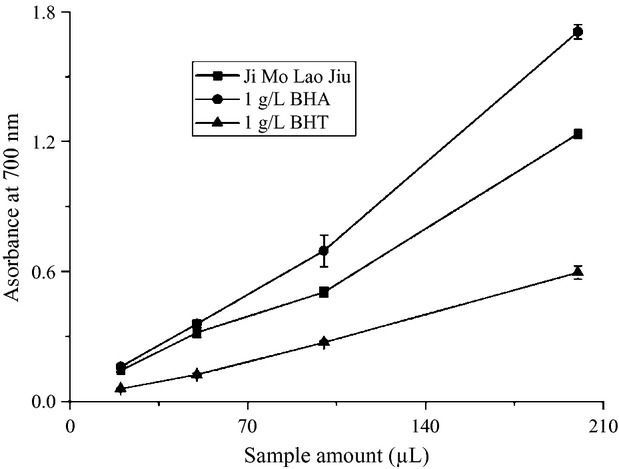
Reducing capacity of Ji Mo Lao Jiu, butylated hydroxyanisole (BHA), and butylated hydroxytoluene (BHT).

The antioxidant activities of natural components might have a reciprocal correlation with their reducing capacity (Duh and Yen 1997; Gülçın et al. 2003). In this study, the Fe^3+^−Fe^2+^ transformation was determined as reducing capacity. The results demonstrated that JMLJ exhibited effective reducing capacity at all amounts. The reducing capacity increased with the increase of JMLJ amount (Fig. 2), indicating some compounds in rice wines were electron donors and could react with free radicals to convert them into more stable products and terminate the radical chain reactions. At the amount of 200 μL, the absorbance of JMLJ, BHA, and BHT at 700 nm was 1.236 ± 0.021, 1.708 ± 0.034, and 0.595 ± 0.031, respectively, which indicated that the reducing capability followed the order BHA>JMLJ>BHT. Both the DPPH radical scavenging activity and the reducing capacity proved that JMLJ had strong antioxidant activities.

## Conclusions

An array of functional components and strong antioxidant activities in terms of DPPH radical scavenging activity and reducing capacity were detected from JMLJ. As previous studies had only detected 16–21 free amino acids (Guang-fa 2008; Zhang et al. 2009; Shen et al. 2010), the present study is the first of its kind in detecting as many as 28 free amino acids in Chinese rice wine, including amino acids with special healthcare functions, such as GABA and ornithine. The large number of functional oligosaccharides, phenolic compounds and mineral elements contributed to the health-promoting effects of JMLJ as well. However, the components responsible for the antioxidant activities of JMLJ are currently unclear. Therefore, further work should be performed on the isolation and identification of the antioxidant components in JMLJ.
